# Linking Global Youth Tobacco Survey (GYTS) data to the WHO Framework Convention on Tobacco Control (FCTC): The case for Tunisia

**DOI:** 10.18332/tid/143994

**Published:** 2022-01-28

**Authors:** Yosr Ayedi, Chahida Harizi, Afef Skhiri, Radhouane Fakhfakh

**Affiliations:** 1 Department of Epidemiology and Biostatistics, Abderrahmen Mami University Hospital, Ariana, Tunisia; 2 Faculty of Medicine of Tunis, University of Tunis El Manar, Tunis, Tunisia

**Keywords:** epidemiology, legislation, cessation, youth tobacco use, tobacco control

## Abstract

**INTRODUCTION:**

The World Health Organization (WHO) had launched the Framework convention on Tobacco Control (FCTC) in 2003 in order to curve the epidemic of tobacco use worldwide. Since most smokers begin to smoke before the age of 18 years, Global Youth Tobacco Survey (GYTS) has been developed in order to monitor tobacco smoking among adolescents. Our aim was to assess smoking among Tunisian youth using GYTS 2017 data.

**METHODS:**

GYTS is cross-sectional, two cluster school-based survey to produce a representative sample of students aged 13–15 years. It was conducted in 2017 in 67 secondary schools in Tunisia. The investigation tool was an anonymously answered questionnaire, which contained core questions about six majors tobacco related topics.

**RESULTS:**

Lifetime cigarettes and waterpipe prevalence were 7.8% (14.4% of boys, 1.6% of girls, p<0.001) and 7.2% (13% of boys, 2.8% of girls, p<0.001), respectively. Among cigarette smokers, 62.5% were able to buy their own cigarettes. Overall, 23.5% of cigarette smokers and 41.5% of waterpipe smokers were not able to buy their products because of their age. Sixty percent of smokers wanted to quit and 56.4% had already tried to stop. Half of the respondents were exposed to SHS in their homes and 62.1% in indoor public places.

**CONCLUSIONS:**

In Tunisia, tobacco prevalence among youth is high. Youth have free access to tobacco products and smoke-free regulations are only partially respected.

## INTRODUCTION

Tobacco use is the first cause of avoidable death and disability around the world^1-3^. It kills more than half of those who smoke regularly^4^. Studies predict that decreasing smoking prevalence up to 15% would reduce mortality due to cardiovascular diseases by up to 7.2% by 2030^5^. Recognizing that the spread of the tobacco epidemic is a serious problem with devastating consequences on health, society and environment, the World Health Organization (WHO) launched the Framework Convention on Tobacco Control (FCTC) in order to curb this epidemic^6^. The FCTC is the first public health treaty on tobacco control; it became an international law on February 2005. It had encouraged all member states to implement a tobacco control and surveillance programs (MPOWER package), such as restricting exposure to second hand smoke (SHS), improving restriction laws on the sale of tobacco products, and working on limiting advertising of tobacco products.

Tobacco dependence commences in adolescence, as most regular adult smokers begin to smoke before the age of 18 years, leading to higher nicotine dependence and adverse outcomes^7,8^. Concerned by the large prevalence of tobacco use among teenagers, mostly in low- and middle-income countries^9^, WHO developed the Global Tobacco Surveillance System (GTSS). The Global Youth Tobacco Survey (GYTS) is one of four surveys of this frame. It is a multinational school-based survey of students in grades associated with 13 to 15 years of age, since early initiation of tobacco may be associated with educational level, socioeconomic status, and family and school affluence^10^. GYTS has been conducted 491 times worldwide until 2018 using the standard methodology of the Center of Diseases Control (CDC) of Atlanta^11^. Tunisia, among the countries of the Middle East and North Africa (MENA) region, has the highest tobacco prevalence among men aged ≥15 years according to WHO estimations. This prevalence is estimated to be 49.3% (95% CI: 36.5–62.9) while it is rapidly increasing among women and adolescents^12^. GYTS is a national survey and has been conducted four times in Tunisia, in 2001, 2007, 2010 and 2017. In this study, we aim to use the data of GYTS Tunisia 2017 to outline the situation of smoking experimentation among youth and to parallel with implemented FCTC articles.

## METHODS

### Data collection

The GYTS is a cross-sectional school-based survey that uses a two-stage cluster sample design to produce a representative sample of students aged 13–15 years. In Tunisia, in the first stage, the list of all public middle schools was sent to the CDC Atlanta where 67 schools from the 24 cities were chosen randomly. The probability of a school being selected is in proportion to the number of students enrolled in the specified grades. In the second stage, one or two classes by school were selected arbitrarily, according to the city population and size. In total, 100 classes were included in this survey. The study was conducted in April and May, 2017. Pollsters were physicians and nurses of a school of medicine, under the coordination of the direction of school and university medicine. Students at this age in Tunisia belong to the 7th, 8th and 9th grades. Each student attending class in the day of the survey was eligible to participate in the study. Questionnaires were anonymously answered. They were first collected in regional administrations, and then sent to the central administration in the Public Health Ministry where data were analyzed.

### Investigation tool

The GYTS survey uses a standard methodology and protocol approved by CDC Atlanta. The questionnaire was validated by CDC and WHO experts^13^. It contained core questions about major tobacco concerns focusing on prevalence of smoked and smokeless tobacco among teenagers attending public schools, their access to different tobacco products, and their desire to quit smoking, their exposure to media and advertising, and to secondhand smoke (SHS). It was translated to Arabic and then send to CDC for further checking to ensure accuracy and reliability. It was first pretested with a focus group of adolescents to ensure that the translation was pertinent and precise.

### Statistical analysis

Data were analyzed using Statistical Package for the Social Sciences (IBM SPSS Statistics version 20). Adjusted and weighting factors were applied to each student record to adjust according to probability of selection and to non-response (by school, class and student).

The weighting factor was: w = w1×w2×f1×f2×f3×f4, with w1 = the reverse of probability of selection of school; w2 = the reverse of probability of selection of class among school; f1 = adjustment factor of non-response among school according to size (large, medium, small); f2 = adjustment factor of class calculated by school; f3 = adjustment factor of student’s non-response calculated among class; and f4 = adjustment factor post stratification calculated by gender and by grade. Analysis of categorical variables was conducted through chi-squared test, and 95% confidence intervals (95% CI) are reported. A two-sided 5% significance level was used for all statistics.

## RESULTS

The response rate was 92.8%. Of 2448 eligible students successfully participating in the survey, 1863 students were aged 13–15 years.

### Prevalence

One in four students (23.5%; 95% CI: 21.6–25.4) had ever tried to use a cigarette, even one or two puffs. Whereas 12.7% of students (95% CI: 11.2–14.2) had ever tried any other smoked tobacco. Meanwhile, lifetime prevalence of smoked and smokeless tobacco was 11.9% (95% CI: 10.4–13.4) and lifetime cigarettes and waterpipe use were 7.8% (95% CI: 6.6–9.0) and 7.2% (95% CI: 6.0–8.4), respectively.

One third of the boys have ever tried to smoke a cigarette (37.4%; 95% CI: 35.2–39.6) compared with 10% of girls (95% CI: 8.6–11.4) (p<0.001). Twenty-one percent of the boys had tried to use waterpipe in their lives (21.3%; 95% CI: 19.4–23.2) versus 6.2% of the girls (95% CI: 5.1–7.3). As for lifetime prevalence, one-fifth of the boys was a lifetime user of any tobacco product (19.4%; 95% CI: 17.6–21.2) versus 4.8% (95% CI: 3.8–5.8) of girls (p<0.001). Boys were significantly more users of cigarettes than girls (14.4%; 95% CI: 12.8–16.0) versus 1.6% (95% CI: 1.0–2.2) (p<0.001), and for waterpipe (13%; 95% CI: 11.5–14.5) versus 2.8% (95% CI: 2.1–3.5) (Table 1).

**Table 1:** Prevalence of tobacco use, by sex, GYTS Tunisia 2017

*Tobacco use*	*Adjusted number*	*Total % (95% CI)*	*Boys % (95% CI)*	*Girls % (95% CI)*	*p*
Current tobacco smoker (smoked and smokeless)^a^	1792	11.9 (10.4–13.4)	19.4 (17.6–21.2)	4.8 (3.8–5.8)	<0.001
Ever tried a cigarette, even 1 or 2 snuffs	1825	23.5 (21.6–25.4)	37.4 (35.2–39.6)	10.0 (8.6–11.4)	<0.001
Lifetime cigarette prevalence^b^	1799	7.8 (6.6–9.0)	14.4 (12.8 – 16.0)	1.6 (1.0–2.2)	<0.001
Frequent cigarette smoker^c^	1859	2.2 (1.5–2.9)	4.2 (3.3–5.1)	0.1 (0–0.002)	<0.001
Ever tried waterpipe smoking	1848	12.7 (11.2–14.2)	21.3 (19.4–23.2)	6.2 (5.1–7.3)	NS
Current waterpipe smoker^d^	1859	7.2 (6.0–8.4)	13.0 (11.5–14.5)	2.8 (2.1–3.5)	<0.001

a Smoked cigarettes or other type of tobacco anytime during the past 30 days.

b Smoked cigarettes anytime during the past 30 days.

c Smoked cigarettes on 20 or more days of the past 30 days.

d Smoked waterpipe anytime during the past 30 days. NS: not significant.

### Access to cigarettes and water pipe

One-third of cigarette smokers (37.6%; 95% CI: 35.4–39.8) got their cigarettes from a shop or a store. Half (50%; 95% CI: 47.7–52.3) bought their cigarettes in a packet and 39.4% (95% CI: 37.2–41.6) had purchased individual cigarettes. Twenty-three percent of cigarette smokers (23.2%; 95% CI: 21.3–25.1) and 41.6% (95% CI: 39.4–43.8) of waterpipe smokers were prevented from buying their own cigarettes and waterpipe due to their age, respectively (Table 2).

**Table 2:** Access and procurement of smokers to cigarettes and waterpipe, by sex, Tunisia, GYTS 2017

*Current smokers*	*Adjusted number*	*Total % (95% CI)*	*Boys % (95% CI)*	*Girls % (95% CI)*	*p*
**Source of cigarettes**					
A shop or store	173	37.6 (35.4–39.8)	40.1 (37.9–42.3)	23.1 (21.2–25.0)	NS
A street seller	174	6.9 (5.7–8.0)	7.4 (6.2–8.6)	3.8 (2.9–4.7)	NS
Gas station	174	18.4 (16.6–20.2)	18.4 (16.6–20.2)	18.5 (16.7–20.3)	NS
Someone else	173	29.5 (27.4–31.6)	27.9 (25.9–30.0)	38.5 (36.3–40.7)	NS
Another way	174	8.6 (7.3–9.9)	6.8 (5.7–7.9)	19.2 (17.4–21.0)	0.037
**Cigarette procurement**					
In a packet	208	50.0 (47.7–52.3)	47.4 (45.1–49.7)	63.6 (61.4–65.8)	NS
In individual sticks	208	39.4 (37.2–41.6)	41.7 (39.5–43.9)	27.3 (25.3–29.3)	NS
In a carton	208	5.8 (4.7–6.9)	6.3 (5.2–7.4)	3.0 (2.2–3.8)	NS
In form of tobacco dust	208	4.8 (3.8–5.8)	4.6 (3.6–5.6)	6.1 (5.0–7.2)	NS
Prevented from buying cigarettes because of age	241	23.2 (21.3–25.1)	22.3 (20.4–24.0)	27.1 (25.1–29.1)	NS
Prevented from buying waterpipe because of age	149	41.6 (39.4–43.8)	36.5 (34.3–38.7)	45.3 (43.0–47.6)	NS

NS: not significant.

### Smoke cessation

Sixty percent (60.5%; 95% CI: 58.3–62.7) of actual smokers had wanted to quit while 56.6% (95% CI: 54.3–58.8) had already tried to stop smoking cigarettes. Seventy-two percent of adolescent smokers had asked for help to quit (72.4%; 95% CI: 70.4–74.4). As for waterpipe, half of the adolescent smokers (44.7%; 95% CI: 42.4–46.9) hoped to quit while 47.3% (95% CI: 45.0–49.6) had already tried to quit.

Boys were more likely to want to quit and to tried quitting more than girls [66.4% (95% CI: 64.3–68.5) vs 23.5% (95% CI: 21.6–25.4); p<0.001] and [61.6% (95% CI: 59.4–63.8) vs 23.8% (95% CI: 21.9–25.7); p<0.001)], respectively. Boys asked for help for cessation more than girls [76.2% (95% CI: 74.3–78.1) vs 53.8% (95% CI: 51.5–56.1); p<0.001] (Table 3). This help had been asked from a professional in 17.9% of the cases.

**Table 3:** Smoke cessation attempts of actual smokers, by sex, GYTS 2017, Tunisia

	*Adjusted number*	*Total % (95% CI)*	*Boys % (95% CI)*	*Girls % (95% CI)*	*p*
**Current cigarette smokers**					
Who want to quit smoking	124	60.5 (58.3–62.7)	66.4 (64.3–68.5)	23.5 (21.6–25.4)	<0.001
Who have tried to quit	159	56.6 (54.1–58.6)	61.6 (59.4–63.8)	23.8 (21.9–25.7)	<0.001
**Current waterpipe smokers**					
Who want to quit smoking	114	44.7 (42.4–46.9)	50.0 (47.7–52.3)	39.7 (37.5–41.9)	NS
Who have tried to quit	150	47.3 (45.0–49.6)	47.1 (44.8–49.4)	47.6 (45.3–49.9)	NS
**Current smokers (cigarettes and waterpipe)**					
Who asked for help	312	72.4 (70.4–74.4)	76.2 (74.3–78.1)	53.8 (51.5–56.1)	<0.001
Who asked a professional for help	312	17.9 (16.1–19.6)	19.2 (17.4–21.0)	11.5 (10.0–12.9)	NS
Who asked family and friends for help	312	63.8 (61.6–66.0)	66.9 (64.8–69.0)	48.1 (45.8–50.4)	0.018

NS: not significant.

### Exposure to SHS, media, and advertising

Almost half of the students (46.7%; 95% CI: 44.4–49.0) were exposed to SHS in their homes during the week prior to the survey, whereas 62.1% (95% CI: 60.0–64.3) and 60.2% (95% CI: 58.0–62.4) were exposed to SHS in indoor and outdoor public places, respectively. Three-quarters (74.0%; 95% CI: 72.0–76.0) reported that they prefer a smoking restriction in indoor public places and 65.2% (95% CI: 63.0–67.4) at outdoor public places. Exposure to SHS was more frequent among boys than girls in outdoor places [62.9% (95% CI: 60.7–65.1) vs 57.7% (95% CI: 55.4–60.0); p<0.05].

As for media and advertising, 69.6% (95% CI: 67.5–71.7) have seen anti-tobacco messages on billboards, newspapers, TV, magazines, and movies. Sixty percent [62.9% (95% CI: 60.7–65.1)] and 32.7% (95% CI: 30.6–34.8) have noticed health warnings on cigarette and waterpipe packages, respectively. Girls were less likely to be exposed to anti-tobacco messages than boys [69.1% (95% CI: 67.0–71.2) vs 72.3% (95% CI: 0.3–74.3); p<0.05], whereas males were more attracted by health warnings on cigarettes packages than females [66.4% (95% CI: 64.3–68.5) vs 59.4% (95% CI: 57.2–61.6); p<0.05] (Table 4).

**Table 4:** Exposure to SHS, to media and advertising, by sex, Tunisia, GYTS 2017

	*Adjusted number*	*Total % (95% CI)*	*Boys % (95% CI)*	*Girls % (95% CI)*	*p*
**Exposure to SHS in the past 7 days**					
Inside home	1858	46.7 (44.4–49.0)	46.3 (44.0–48.6)	47.1 (44.8–49.4)	NS
In public indoor places	1859	62.1 (60.0–64.3)	63.7 (61.5–65.9)	60.6 (58.4–62.8)	NS
At public outdoor places	1858	60.2 (58.0–62.4)	62.9 (60.7–65.1)	57.7 (55.4–60.0)	0.022
Inside school	1847	78.6 (76.7–80.5)	78.2 (76.3–80.1)	78.9 (77.0–80.8)	NS
In favor of smoking ban					
In indoor public places	1842	74.0 (72.0–76.0)	73.8 (71.8–75.8)	74.3 (72.3–76.3)	NS
At outdoor public places	1830	65.2 (63.0–67.4)	63.1 (60.9–65.3)	67.3 (65.2–69.4)	NS
**Exposure to media and advertising in the past 30 days**					
Anti-tobacco media messages	1859	69.6 (67.5–71.7)	66.9 (64.8–69.0)	72.3 (70.3–74.3)	0.012
Health warnings on cigarette packages	1859	62.9 (60.7–65.1)	66.4 (64.3–68.5)	59.4 (57.2–61.6)	0.002
Health warnings on waterpipe packages	1859	32.7 (30.6–34.8)	33.6 (31.4–35.7)	31.7 (29.6–33.8)	NS
Told about smoke dangers in school	1844	50.4 (48.1–52.7)	45.8 (43.5–48.1)	54.8 (52.3–57.1)	<0.001
Advertisements or promotions for tobacco products at points-of-sale	1859	43.7 (41.4–45.9)	47.4 (45.1–49.7)	39.8 (37.6–42.0)	0.003

SHS: secondhand smoke. NS: not significant.

## DISCUSSION

Article 20 of the WHO FCTC calls countries to put in place national and regional plans of action for the supervision of determinants and consequences of tobacco use among adults and adolescents. GYTS is one of the important and dynamic monitoring procedures to reach this goal. It has focused on the measure of seven tobacco related topics, including tobacco prevalence, access of minors to tobacco products, exposure to SHS, intention to smoke, cessation, media and advertising, and school programs on tobacco control.

Tunisia is a middle-income country (MIC), which belongs to the WHO-EMRO. It ratified the FCTC on 7 June 2010^14^. Almost 8% of Tunisian students were current cigarette users. One in six boys was a smoker. Our prevalence remains among the highest in Africa, for both genders combined, and for boys as well^15^. In 2010, 6.6% of Tunisian teenagers were cigarette smokers (12.4% of boys and 2.4% of girls)^16^. A significant decrease in prevalence was expected after the adoption of the WHO FCTC. Nevertheless, instead, we found a neglegible increase from 2010 to 2017, which may be explained by a total release of all tobacco prevention and control actions^17^.

As for waterpipe use, over 7% of our students were smokers. Waterpipe is an epidemic in the MENA region; it had surpassed cigarettes in terms of popularity. A meta-analysis published in 2014 showed the prevalence ranged from 2% in Oman in 2010 to 35% in Lebanon in 2011. Other countries in the world have been witnessing a great increase also, such as UK and US, where narghile exceeded conventional cigarette use^15^.

### Access to cigarettes and water pipe

Article 16 of the WHO FCTC requires signing members to implement effective and legislative measures for interdiction of all form tobacco sales to minors (aged <18 years).

In the month prior to the survey, 62.9% of cigarette smokers were able to buy their own cigarettes. Almost one-quarter of cigarette smokers and 41.6% of waterpipe users were prevented from buying their products due to their age, respectively. The National Youth Tobacco Survey (NYTS) in the US conducted from 2016 to 2018 among teenagers aged 9–17 years (middle school and high school) found that 15.6% had bought their own cigarettes in 2016. This rate had decreased to 11.4% in 2018, with a significant difference between the 2 years. The refusal rate of tobacco products sales because of age in the American study was almost constant (24.7% in 2016 and 25.5% in 2018)^18^. Results of GYTS conducted in Italy in 2010 and 2014 among 1500 teenagers showed a significant 22% decrease in adolescents buying their cigarettes from a point-of-sale not a vending machine (from 48.8% in 2010 to 38.8% in 2014). The same study showed a significant improvement in refusing to give tobacco products to minors by 30% (91.7% in 2010 to 63.9% in 2014)^19^. These results were the opposite of ours, which may be due to the early implementation of laws prohibiting tobacco products sales to the underage in the US since 1992 and the enforcement of existing restricting rules in Italy in 2013^19^. Thus, in Tunisia, we have not signed yet any law that restricts selling tobacco products to minors^14^. It is highly recommended that such a law is implemented since it has an important role in decreasing smoking prevalence among youth and denormalizing cigarette buying by adolescents^20^.

### Smoking cessation

Article 14 encourages countries to establish multiple reduction measures concerning tobacco dependence and cessation. We found in our study that 60.5% of smokers had wanted to quit smoking and 56.6% had already tried to quit. Seventy-two percent had asked for help to quit. A meta-analysis was performed in 2019 including 11 international studies from five middle- and high-income countries conducted among 6469 current or former smokers over the last 20 years, using the Trans-theoretical method^21^. It found that the prevalence of the desire to quit smoking (C and P stages) varied from 6% in the Czech Republic to 31.1% in Turkey, and the prevalence of smokers attempting to quit (A stage) varied from 13.2% in 2001 to 28% in 2012 in the US. Gorini et al.^19^ found that the desire to stop smoking increased from 28% in 2010 to 43.5% in 2014 (55% increase). Sixty percent of smokers confirmed they had already tried to quit. In Tunisia, in 2007, the desire to quit smoking was 84% while the prevalence of attempts to stop was 71.4%^22^, which decreased to 52.2% in 2010^16^. Almost three-quarters of students had asked for help to quit. In one in five times, this help was asked from a professional. According to the NYTS in the US in 2013, adolescents who had received advice to stop smoking from a parent were more likely to attempt quitting^23^. Programs helping adolescents to stop smoking should motivate them to quit and raise their awareness about the potential harms. Implementing a national program designated to help teenagers is necessary in Tunisia to support them, since it is harder to quit among adolescents. They are more vulnerable to the desire of smoking and believe the negative effects are long-term effects. In addition, giving nicotine substitutes is prohibited before the age of 17 years^23^.

### Exposure to SHS

FCTC Article 8 calls member states to adopt in places where people are gathered, such as public indoor places, public transport and indoor workplaces, legislative and executive laws that prohibit smoking to protect non-smokers from exposure to SHS.

In the week prior to the survey, 46.7% and 62.1% of the students were exposed to SHS in their homes and in public indoor places, respectively. Three-quarters were in favor of a smoking ban in public indoor places. A meta-analysis conducted in 38 different LMIC countries from 2003 to 2016 had included 88209 non-smoker adolescents aged 12–15 years. It had shown that the overall prevalence daily and non-daily exposures to SHS were 15.7% and 34.2%, respectively. These rates varied from 2.6% in Cambodia to 35.4% in Indonesia; and from 19.8% in Bangladesh to 56.3% in Vietnam, respectively^24^. In 2017, according to the NYTS, 13.2 million youth (50.5%) reported exposure to SHS in a public place or more. The last public smoke-free environment law before the survey was implemented in the US in 2010, thus, millions of American teenagers continue to reveal their exposure to different tobacco products^25^. In Tunisia, the previous GYTS survey of 2010 concluded that 49.9% and 38.6% of the students were exposed to SHS inside and outside their homes, respectively^16^. The first implemented law in terms of tobacco control was law n° 98-17 of 23 February 1998, which protects people from tobacco harmful effects^26^. It had been enforced by decree n°98–2248, which had listed the places where it was forbidden to smoke. These places cover schools, lecture rooms, entertainment and sports locations, especially those hosting minors^27^. Then, decree n°2009–2611 was adopted defining smoking areas’ plans^28^. However, violation rate of these laws is still so high in Tunisia since the majority of smokers have continued to smoke in smoke-free places^14^.

### Exposure to media and advertising

FCTC Article 13 asks member states to a comprehensive ban, or if not possible, restriction of all forms of tobacco advertising, promotion and sponsorship.

In our survey, 43.7% of teenagers reported being exposed to publicity and promotion for tobacco products in points-of-sale. A meta-analysis published in 2016 that included more than 180000 teens aged 9–18 years showed that children and teenagers exposed frequently to point-of-sale (POS) tobacco promotion were 1.6 and 1.3 times more likely to try smoking and to start smoking, respectively. It confirmed that exposure to advertisements and promotion at POS is a significant risk factor to youth smoking^29^. The European Survey Project on Alcohol and Other Drugs (ESPAD) conducted in 25 European countries among adolescents aged 15–16 years in 2007, 2011 and 2015 found that the POS display ban implemented in Europe had decreased regular smoking odds by at least 15%^30^. In Tunisia, there is a comprehensive ban of tobacco promotion in media, but not in POS^14^. GYTS 2010 found that almost 60% of the students were exposed to POS promotion at least a few times during the month prior to the survey^16^. It is, however, recommended to strengthen enforcements and bans of promotion and sponsorships, especially in POS and school neighborhoods since these restrictions may decrease smoking prevalence by at least 16% by 2065^31^.

### Strengths and limitations

One of the strengths of our survey is that it is a national representative sample, which is, to our knowledge, the first national survey since 2010. In addition, GYTS has been conducted in 185 countries around the world using the same questionnaire (with a few differences related to the countries) and the same methodology of the CDC Atlanta. Nevertheless, it has some limitations. First, it did not report the city and the place of the candidate, which did not allow us to have a specific idea about tobacco use and different exposures among big versus small cities, or urban versus rural regions. Second, the survey presented a selection bias. In fact, the sample was only taken from public school students, without taking into consideration private school students or those who had quit school early. Thus, public school students represented only 10% of Tunisian adolescents according to the UNESCO and quitting school at an early age may be associated with higher prevalence of tobacco use^32^. Although GYTS had been previously conducted in Tunisia, reliability and validity measures have not been tested and the Tunisian version of the questionnaire had not been validated^33,34^.

### Recommendations

We highly recommend that the law prohibiting tobacco products sales to minors should be signed. The country needs to implement effective surveillance methods to monitor tobacco use in smoke-free places. It also must control POS promotions. Moreover, Tunisia has not conformed to WHO guidelines in terms of taxation system. FCTC Article 6 calls countries to implement prices policies in order to reduce tobacco demand and consumption. The last increase in tobacco prices was in 2020 but it was not due to taxation. Tunisia has dropped into a lower level in terms of convenient taxation system in 2019^35^. The financial system of cigarettes should be revised to achieve a targeted decrease in tobacco use.

## CONCLUSIONS

WHO had launched the FCTC to curb the epidemic of tobacco worldwide. GYTS came in synergy with the objectives of the convention. It gave countries the occasion to judge their compliance with the goals of the FCTC and to evaluate their laws in terms of tobacco control. Tunisia is currently not in a good position. Tobacco prevalence is alarming, youth have free access to tobacco products and smokefree regulations are only partially respected. Actions are needed to strengthen tobacco control measures targeted at adolescents in Tunisia.

## Data Availability

The data supporting this research are available from the authors on reasonable request.
